# LLAD: Life-Log Anomaly Detection Based on Recurrent Neural Network LSTM

**DOI:** 10.1155/2021/8829403

**Published:** 2021-02-24

**Authors:** Ermal Elbasani, Jeong-Dong Kim

**Affiliations:** ^1^Department of Computer Science and Engineering, Sun Moon University, Asan 31460, Republic of Korea; ^2^Genome-based BioIT Convergence Institute, Sun Moon University, Asan 31460, Republic of Korea

## Abstract

Life-Log is a term used for the daily monitoring of health conditions and recognizing anomalies from data generated by sensor devices. The development of smart sensors enables collection of health data, which can be considered as a solution to risks associated with personal healthcare by raising awareness regarding health conditions and wellness. Therefore, Life-Log analysis methods are important for real-life monitoring and anomaly detection. This study proposes a method for the improvement and combination of previous methods and techniques in similar fields to detect anomalies in health log data generated by various sensors. Recurrent neural networks with long short-term memory units are used for analyzing the Life-Log data. The results indicate that the proposed model performs more effectively than conventional health data analysis methods, and the proposed approach can yield a satisfactory accuracy in anomaly detection.

## 1. Introduction

Healthcare and wellness have garnered significant attention in the last two decades from scholars and researchers in all fields of technology and science. Big data in healthcare is crucial for addressing issues associated with the amount of data generated. Technological development has increased the quality of wearable sensor devices, enabling the generation of various data for monitoring health conditions. Regardless of the clinical methods for monitoring health conditions, the current technology enables a significant amount of data to be received to obtain information regarding acute and chronic conditions. Wearable smart devices are increasingly being used in healthcare solutions [1]. These devices integrate the functionalities of traditional medical devices to record medical data [2] such as blood pressure, heart rate, galvanic skin response (GSR), and temperature as well as data regarding physical activities such as walking, running, and sitting. The data are then aggregated into a system to be processed and analyzed for the required applications. Life-logging is a process of recording personal data related to all individual activities as well as interactions with the physical and virtual environments [3, 4]. The data generated from smart devices or applications are in time-series format, in which information is recorded for each specific time. Anomalies in log data refer to certain patterns or points in data that deviate from average data [5]. Techniques for anomaly detection have been applied to various systems. Many methods and algorithms have been designed for anomaly detection, including conventional statistical methods and machine learning with supervised and unsupervised methods [6, 7]. However, these aforementioned methods cannot correctly identify anomalies or perform analysis based on extracted data features; hence, more complex approaches have been developed. Recently, deep learning methods have been used in anomaly detection and log analysis to improve accuracy and achieve higher automation. Among neural networks, those with a high performance in time-series data are recurrent neural networks (RNNs) and long short-term memory (LSTM) [8, 9].

An RNN–LSTM-based model known as Life-Log Anomaly Detection (LLAD) is proposed herein to effectively detect anomalies in health log data aggregated from several devices. Because RNN–LSTM demonstrates a good performance with respect to multivariate sequential data, the same data are processed to be analyzed for anomaly detection using a multivariate RNN–LSTM approach. The methods are compared in terms of proficiency in providing higher accuracy results for detection of health condition.

The remainder of this paper is organized as follows.  Section 2 presents the background and related studies.  Section 3 describes the anomaly detection methods and the characteristics of the data used for the experiments. The experimental results are presented and discussed in Sections 4–6.  Section 7 provides the conclusion and future work.

## 2. Background and Related Studies

This research is categorized under the field of merging of healthcare data analysis and LSTM for anomaly detection. Recently, there have been an increasing number of deep learning approaches for detecting anomalies. A comprehensive set of information for general applications can be found in [10].

### 2.1. Anomaly Detection in Healthcare

Several machine learning approaches that use automatic detection based on electrocardiogram (ECG) abnormal features for binary classes have been proposed [9, 11, 12]. However, these techniques do not include overall health monitoring at a high level of accuracy. Another drawback is that anomalies that are not annotated manually in training data or novelties may not be detected. Applying deep learning has yielded better performance. The detection of critical points in medical data has been performed for leukemia and other chronic diseases [13]. A significant challenge regarding feature extraction in health data is the accurate detection of anomalies. Several methods have been developed for modeling sequential data based on time-series for modeling data, such as linear dynamic systems and the hidden Markov model [14, 15].

The RNN is the latest model for sequential data fraud detection. Most studies regarding anomaly detection in the healthcare domain focus on ECG based on feature extraction and time-series [11]. RNN–LSTM uses data points and prediction error likelihood for detecting anomalies [16]. The LSTM encoder-decoder anomaly detection method is applied for ECG data where the encoder, after learning from a sequence, can reconstruct the sequence to achieve better predictions [17]. Another deep learning method based on feature extraction is convolution neural network (CNN), which can address high-dimensional data more rapidly [18].

### 2.2. Log Anomaly Detection Based on RNN-LSTM

Recently, RNN-LSTM has been used extensively for log data analysis, based on the similarity of LSTM methods used in natural language processing [19, 20]. The clustering method [21] is used for multiple log entries that are input to the LSTM network for detection and prediction of system failure. A generalized detection and diagnosis based on LSTM is used when raw data are parsed and then analyzed for detection [22]. Stacked LSTM is a deep architecture used in log data, where the output of each LSTM layer is an input for the next LSTM layer, and the recurrent layer in time can be unfolded as a feedforward network [4]. Compared with the conventional RNN, LSTM requires minimal or no data preprocessing; furthermore, it does not require features prepared by experts as it operates on raw data, nor does it require prior annotation for anomalies to function.

RNN-LSTM can perform multivariate sequential time-series to detect fraud points in latent features, without the need for dimensionality reduction. In some studies, LSTM utilizes a multivariate Gaussian distribution [18]. Some approaches are similar to the proposed solution [8, 12] in that LSTM is used for anomaly detection; however, in general, nonspecific studies have provided results based on health data analysis from multiple log data sources, such as LSTM time-series modeling. Most health research data focus primarily on ECG. In multivariate cases, annotation is required, and issues such as disbalanced data must be addressed, as detecting anomalies from sequential log format includes learning long-term dependencies that contribute to the final detection performance.

## 3. Proposed Method

This study focused on recognizing abnormal cases in human health conditions, with heart rate (HR) as the main feature for anomaly detection, based on measurements from all sensors. The data results were analyzed using traditional RNN methods and then compared with LSTM. LSTM layers were built with multiple recurrent LSTMs, that is, each LSTM layer output is an input for the next LSTM layer.

### 3.1. Conceptual Model for LLAD

**Figure 1:**
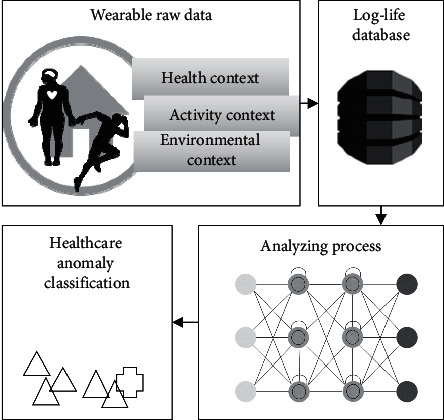
LLAD conceptual model.

The LLAD model used in this study is illustrated in Figure 1. An automated healthcare analysis model for self-monitoring was created in this study to increase attention to well-being. Wearable devices record health signals associated with daily activities and information regarding the surrounding conditions, such as temperature. Subsequently, wireless communication is used to record the data in a database, in which they are synchronized and stored in a log file format. The dataset with health data is known as the Log-Life data. The data are analyzed by RNNs that utilize LSTM, and the results are shown to the user for obtaining information about irregular health states or patterns to prevent the development of nonhealthy stages.

### 3.2. Architecture of LLAD Classification Model Based on RNN-LSTM

Numerous RNN-LSTM architectures have been described and applied for time-series data analysis, and research is being conducted to determine a more efficient method [23]. In addition, many studies have been conducted to determine the most efficient architecture. Whereas some architecture can perform better in specific cases [24], the standard LSTM architecture was used in this study. The LSTM network maps the input sequence *x*_(1:*T*)_*t* = 1 to *T*. Unique embedded vectors for each element in the log data are then mapped to a sequence of hidden vectors *h*_(1:*T*)_ (1) as follows:

(1) $$\begin{array}{c} \text{LSTM}\left(x_{\left(1:T\right)}\right)=h_{\left(1:T\right)}, \end{array}$$

(2) $$\begin{array}{c} p_{\left(t\right)}=softmax\left(h_{\left(t-1\right)}W+b\right), \end{array}$$

where *h* at time *t* is a summary of the input sequence *x*_(1:*T*)_; for the given hidden state at time *t* − 1, the weight *W* and the bias-vector-calculated *p* (*t*) is the probability distribution over sequence at time *t*.

**Figure 2:**
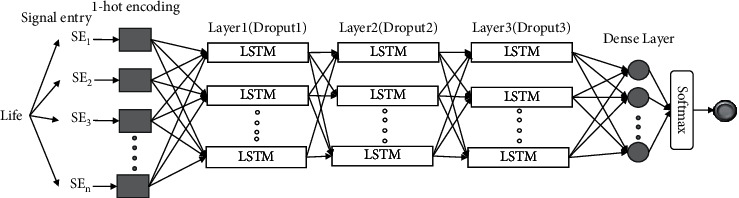
Utilizing log processing for time-series LSTM analysis.

The LLAD classification architecture for analyzing the Life-Log data is shown in Figure 2. The input vector is obtained from 1-hot encoding, which represents the individual signal entries. The encoding yields a dictionary, in which the signal entries and ID that represent a specific code for the entry are associated with each other. A log parser is used to obtain the log ID and values; subsequently, they are organized in matrices. Considering the matrices are based on sensor settings, representing a set with a different execution path, the path of each vector length may differ.

In this study, to analyze Log-Life data, a model comprising three LSTM layers, a single dense activation layer, and a single Softmax output layer was used. Between each layer, a 20% dropout rate was applied. Each of the LSTM layers contained 100 hidden cells.

**Figure 3:**
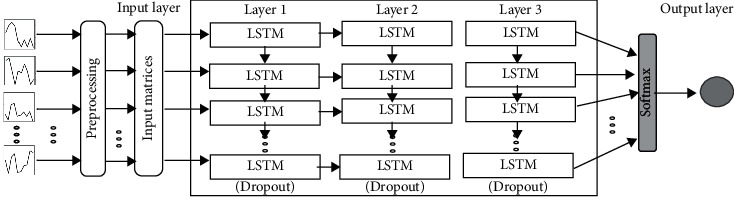
Multivariate data structure modeling.

The same three-layer LSTM structure was used for processing seven data features; herein, this is referred to as the multivariate model (LSTM-MV). The data underwent a standardization process, where the entire data were normalized, converted into a dataset matrix, and then split in training/test sets as input for LSTM (Figure 3).

## 4. Experiment

A number of experiments were conducted to determine the differences in effectiveness between different architectures. This section discusses the data, experimental setup, evaluation metrics, and results from assessing the performances of the proposed methods.

The dataset used in this study was that from [25], which was obtained experimentally from two different devices capturing physical activities in approximately 70 days. The data structure enables the following measurements: HR [bpm] (numeric), steps (numeric), GSR (numeric), burned calories (numeric), skin temperature (°C) (numeric), and date and time (format “YYYY-MM-DD HH:MM:SS”).

**Figure 4:**
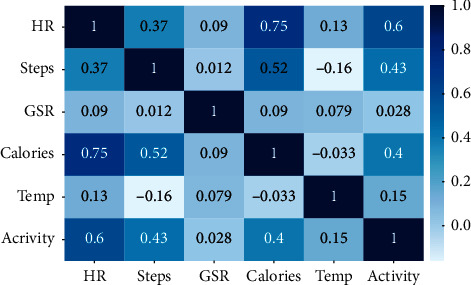
Correlation matrix of all data attributes.

In the preprocessing stage, based on the correlation of feature steps, the HR, GSR, and correct time are calculated by a new feature, which is the tracking of seven activities, that is, standing, running, walking, fast walking, lying, and sleeping.  Figure 4 demonstrates the statistical properties of all the attributes and correlations of the total instances of data with each other. The correlation matrix enables one to identify features that have a greater effect on the target attribute, HR. It is noteworthy that the scale range of the features is distinct and that normalization must be performed such that the attributes are in the same range for efficiently training the neural network. Furthermore, the general attributes have a normal distribution and do not exhibit a strong correlation between them.

**Figure 5:**
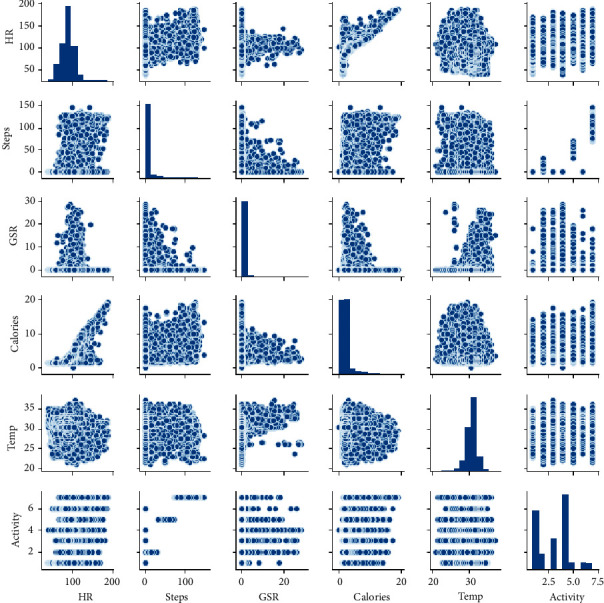
Scatter-plots graphs of all attributes.

The scatter plots of the features depicted in Figure 5 show that the HR has a low positive correlation with the calories, steps, GSR, temperature, and activity, in the descending order. An analysis of each attribute shows that they belonged to the low positive and exhibited almost no strong correlation. In general, this matrix indicates a weak correlation among the attributes; this may not yield multicollinearity in the data, thereby suggesting a high probability of overfitting. The scatter plots show the degree to which one variable is affected by another.

## 5. Results

Based on the data collected, RNN-LSTM was applied based on the multivariate structure and RNN log format analysis. At this stage, these two methods were compared, and their performances were described. To evaluate the performance of machine learning for anomaly detection, the methods were based on matrices expressed in terms of the number of false positives (FP) and false negatives (FN). Standard metrics, such as precision, recall, and *F*-measure, were used. Precision = TP/(TP + FP) (TP denotes true positive) measures the percentage of true anomalies among all anomalies detected. Recall = TP/(TP + FN) measures the percentage of anomalies in the dataset (assuming that the ground-truth is known) being detected, and F-measure = 2 × precision × recall/(precision + recall) is the harmonic mean of the previous two indicators. A study of performance measures for classification tasks that are used widely in learning techniques is presented in [26].

**Figure 6:**
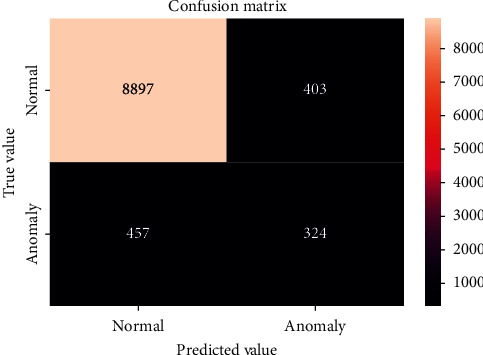
Confusion matrix for the LLDA model.

**Figure 7:**
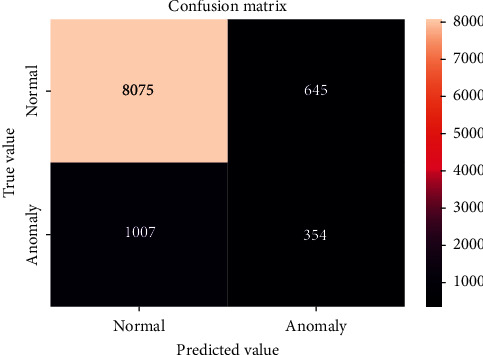
Confusion matrix for LSTM-MV.

The confusion matrices for the LSTM methods are shown in Figures 6 and 7. The *x*- and *y*-axes represent the predicted and true values, respectively. The confusion matrix results show the number of correct and incorrect detections for anomaly detection. The LSTM for the LLAD has a higher rate of correct and incorrect predictions compared with the LSTM-MV. Based on the analysis of both methods, the results of classifying the anomalies in the data are shown in Table 1. First, experiments were performed on 10 days of activities, and the performance of even neural networks was not promising, considering the long-term data collected. In the next step, RNN-LSTM was used on all the data for anomaly detection, and the results are shown in Table 1. Several experiments were performed to achieve the best results based on these parameters. Another test was conducted by analyzing only one variable that used the HR from the data. The likelihood value was lower than the normal range for all data groups. “Exceptional” moves appeared at every point of the abnormal subsequence, although a significant amount of “normal” behavior existed between them. Therefore, a significant proportion of the “abnormal” order was predicted to be exceptional.

## 6. Discussion

Based on the results presented, the LSTM applied in the LLAD analysis performed better than the LSTM-MV analysis method. The precision based on the LLAD method was 96%, whereas that of the LSTM-MV was 92%. Furthermore, it was confirmed that the log data model performed better than the other methods; however, in comparison with cases with the HR feature only, it indicated a lower accuracy. Therefore, it can be concluded that a significant amount of anomaly log system detection does not necessarily yield the best results.

Some limitations were identified in this study. First, the results for CPU loading and execution time were not provided. It can be concluded that LLAD outperformed LSTM-MV by ∼4% in terms of accuracy; however, to determine the overall performance, more experiments may be required. Second, the amount of data used for the experiments was small. Additionally, in both cases, a simple preprocessing of the data before analysis was required. The data were arranged to correspond to the input of each method. To evaluate the effectiveness of the method, a more complex structure should be investigated. In addition, a small number of wearables was used as only seven activities were analyzed.

Analyzing health data in the log format can be more efficient, particularly for technical anomaly detection. Log data tend to differ from databases in terms of complexity, and they require less space to be stored. Analyzing health data for anomaly detection provides a solution for health technologies to support approaches that increase the efficiency of devices providing health maintenance services. RNN with LSTM demonstrated good results in the time-series analysis. It performed better when modeled for the LLAD structure, similar to the single-feature model. Architectures that outperform LSTM on sequential data modeling exist; however, they are not to be considered as methods that perform better consistently in many cases and apply to generalized cases.

Whereas deep learning has widely been considered for a diverse range of applications, few studies have been conducted to investigate deep learning, particularly for LLAD. Anomaly detection is considered as a difficult problem. An anomaly is detected as a deviation from the normal pattern; however, it is difficult to define normality that accounts for every variation in a normal pattern. Therefore, defining anomalies is difficult. Anomalies are rare events, and it is impossible to obtain prior knowledge regarding every anomaly type. Moreover, the definition of anomalies varies across applications. In this study, the term “anomaly data” is used to describe an anomaly related to a health condition. Although anomaly cases in data must be categorized and the extent to which the anomalies represent real anomalies in health must be considered, obtaining a log anomaly system that generalizes all cases is almost impossible because of the features of different log types. In addition, this study not only provides an approach that is directed to specific anomaly types but also diagnoses unknown patterns of anomalies.

## 7. Conclusions

This study provides a new approach for using an RNN that utilizes LSTM to analyze healthcare data. The specific feature of this approach is the log format analysis of health data. The experimental results were promising; the model based on the LSTM structure showed a highly promising 96% for log data structure and 92% on multivariate data, whereas the binary classification accuracy was 97%, based on the HR datasets. This method of health data analysis will render it easier for current technologies to provide efficient analysis when utilizing the potential of deep learning in health data, which is essential in big data.

It was demonstrated in this study that, in log data, the proposed model performed better than the conventional machine learning techniques by providing greater accuracy. Therefore, adapting the previous anomaly detection system for life-logging data is not always the best solution. A case study for an RNN utilizing LSTM was presented herein, and it was discovered that a multivariate model for sequential data can be used for anomaly detection in this scenario. Furthermore, the effects of different parameters and architectures on system performance were discussed. For future studies, a larger dataset with more activation functions should be developed. In addition, several machine learning methods should be implemented and then tested with several datasets for anomaly detection.

## Figures and Tables

**Table 1:** Results of implemented methods' performance for anomaly detection.

Method	Precision (%)	Recall (%)	F-1 measurement (%)
LLAD	96	98	97
LSTM-MV	92	90	89

## Data Availability

The data used to support this study are available on request through contact to the corresponding author.
